# The relationship between mindfulness and objective measures of body awareness: A meta-analysis

**DOI:** 10.1038/s41598-019-53978-6

**Published:** 2019-11-22

**Authors:** Isaac N. Treves, Lawrence Y. Tello, Richard J. Davidson, Simon B. Goldberg

**Affiliations:** 1 Department of Brain and Cognitive Sciences, Massachusetts Institute of Technology, Cambridge, Massachusetts USA; 2 School of Public Health, University of California – Berkeley, Berkeley, CA 94720 USA; 3 Center for Healthy Minds, University of Wisconsin – Madison, Madison, WI 53703 USA; 4 Department of Psychology, University of Wisconsin – Madison, Madison, WI 53706 USA; 5 Department of Counseling Psychology, University of Wisconsin – Madison, Madison, WI 53706 USA

**Keywords:** Predictive markers, Quality of life

## Abstract

Although awareness of bodily sensations is a common mindfulness meditation technique, studies assessing the relationship between mindfulness and body awareness have provided mixed results. The current study sought to meta-analytically examine the relationship between mindfulness operationalized as a dispositional trait or a construct trained through short- (i.e., randomized controlled trials [RCTs]) or long-term mindfulness meditation practice with objective measures of body awareness accuracy. PubMed, Web of Science, PsycINFO, and Scopus were searched. Studies were eligible if they reported the association between mindfulness and body awareness, were published in English, and included adults. Across 15 studies (17 independent samples), a small effect was found linking mindfulness with greater body awareness accuracy (*g* = 0.21 [0.08, 0.34], *N* = 879). When separated by study design, only RCTs continued to show a significant relationship (*g* = 0.20, [0.02, 0.38], *k* = 7, *n* = 505). Heterogeneity of effects was low (*I*^2^ < 25%), although with wide confidence intervals. Effects were not moderated by study quality. Low fail-safe N estimates reduce confidence in the observed effects. Results suggest a small but potentially detectable relationship between mindfulness and body awareness accuracy. Future investigations could examine individual differences in body awareness as a mechanism within mindfulness interventions.

## Introduction

Mindfulness has been defined as accepting, open-minded attention to the present moment^1^. It has been conceptualized by different investigators as both a momentary state^2^ or dispositional trait (i.e., attending mindfully to daily life more generally^3^). Theoretically, mindfulness can be cultivated through training, be it in the form of brief interventions (e.g., eight weeks in mindfulness-based stress reduction [MBSR]^4^) or long-term meditation practice^5^. Many common mindfulness practices involve attending to bodily sensations (e.g., the body scan taught in MBSR that involves moving non-judgmental attention throughout regions of the body). Mindfulness training is theorized to improve body awareness, facilitating access to bodily sensations^6,7^. In turn, body awareness is thought to be a crucial element in self-regulation and decision-making^8–10^. Indeed, many adverse health conditions are characterized by dysfunctions in body awareness (e.g., anorexia, chronic pain, addiction^11–13^).

Accumulated evidence supports the clinical benefits of mindfulness interventions^14,15^ and both dispositional mindfulness and training in mindfulness are associated with well-being in healthy populations^16–18^. Dispositional mindfulness and training in mindfulness have also been linked to greater self-reported body awareness (e.g., using the Multidimensional Assessment of Interoceptive Awareness [MAIA]^19–21^). In addition, mindfulness training has been associated with increased gray matter and functional activation in brain areas involved in body awareness (e.g., insula^22–24^).

Despite some promising evidence connecting mindfulness and body awareness, findings from studies directly examining the association between mindfulness and objective measures of body awareness have been mixed. This is the case for studies examining trait mindfulness^25,26^, the experimental effects of mindfulness training^27,28^, and the relationship between long-term training and objective measures of body awareness^29,30^. Thus, it remains unclear if and how mindfulness actually relates to body awareness.

Whether this link exists is important for several reasons. Objective measures of body awareness, which include tasks like heartbeat tracking or tactile detection^31^, assess a specific aspect of body awareness – accuracy. Body awareness accuracy has been defined as the “correct and precise monitoring” of bodily sensations^32^. By evaluating the relationship between mindfulness and objective measures of body awareness, we test one element within the theory of secular mindfulness (i.e., that mindfulness facilitates access to bodily sensations^4^). Further, a lack of evidence for this link may imply that actual increases in body awareness accuracy are unrelated to benefits of mindfulness. Rather, it may be that improvement in subjective body awareness or changes in the appraisal of bodily sensations (e.g., as assessed via the MAIA^4,6,7,33^) are more closely tied to mindfulness and its potential benefits than objective accuracy.

In the current study we attempt to clarify the relationship between objective measures of body awareness and mindfulness – whether mindfulness is operationalized as a dispositional trait or cultivated through brief training or long-term practice. To do so, we conducted a systematic review and meta-analysis quantifying this relationship. In addition, we explored whether effects varied systematically based on study design (e.g., trait mindfulness vs. experimentally-induced mindfulness) and study quality.

## Results

### Study selection

**Figure 1:**
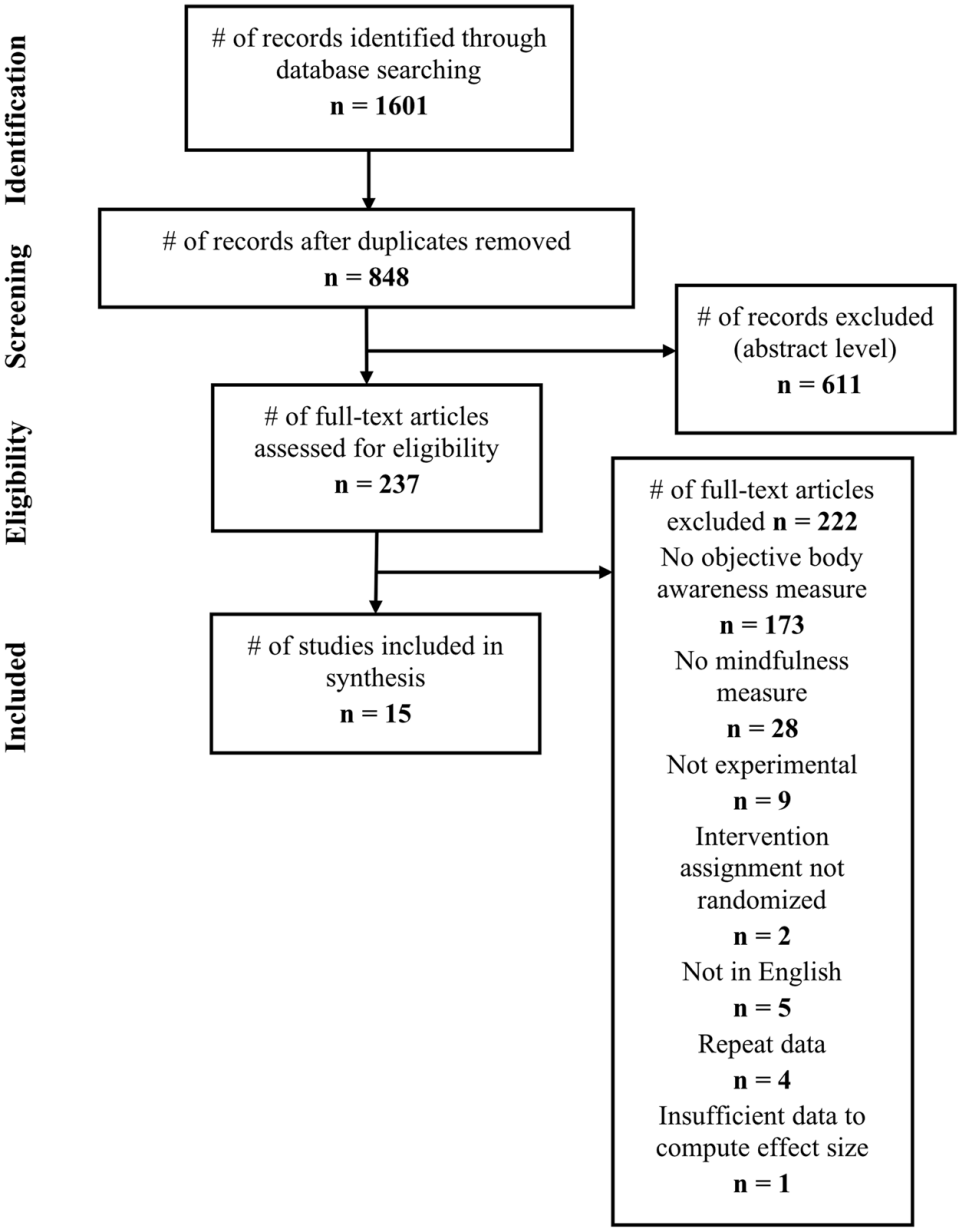
PRISMA flow diagram.

Two successive searches were performed, yielding 1,186 and 415 citations, respectively (Fig. 1). After removing duplicates and excluding based on title and abstract, full texts were reviewed for the remaining 237 studies. The final sample included 15 studies with 17 independent samples representing 879 participants (Table 1).

**Table 1:** Summary of included studies.

Author (date)	Mindfulness design	Body awareness task	Task type	Number of subjects	Subject demographics	Mindfulness type/control	Significant result found?
Cebolla 2016b^*^^42^	Trait	proprioceptive drift	distal	30	38.07 yrs 50% F	FFMQ	No
Kiken 2018^25^	Trait	blood glucose accuracy	proximal	25	55.05 yrs 67% F	SCS-M MAAS	Yes No
Parkin 2014b^26^	Trait	heartbeat tracking	proximal	105	41.28 yrs 72% F	FFMQ	No
Aaron 2017^34^	RCT	heartbeat tracking	proximal	38	19.70 yrs 66% F	10 min BS	No
				38		Story Listening	
Bornemann 2017^27^	RCT	heartbeat tracking	proximal	147	40.69 yrs 60% F	9-month MM	Yes
				79		retest	
Fischer 2017a^35^	RCT	heartbeat tracking	proximal	25 24	22.50 yrs 80% F	8-week BS Story Listening	No
Fischer 2017b^35^	RCT	heartbeat tracking	proximal	18 18	22.50 yrs 100% F	8-week BS inactive	Yes
Mirams 2013^31^	RCT	tactile detection	proximal	31	19.21 yrs 90% F	1-week BS	Yes
				31		Story Listening	
Parkin 2014a^26^	RCT	heartbeat tracking	proximal	20	44.51 yrs 73% F	1-week BS	No
				20		inactive	
Wooten 2018^28^	RCT	joint kinesthesia	distal	6	73.15 yrs 75% F	6-week BS	No
		joint position sense	proximal	10		movement therapy	
Cebolla 2016a*^42^	LTM	proprioceptive drift	distal	15 15	38.07 yrs 50% F	MM (9 yrs) MNP	No
Daubenmeier 2013^36^	LTM	respiratory load (RL) detection	distal	18 16	38.84 yrs 44% F	Vipassana (9 yrs) MNP	Yes
		RL discrimination	distal				
		respiratory tracking	proximal				
Fox 2012^37^	LTM	tactile sensitivity	distal	9	39.5 yrs 50%	Vipassana/BS	Yes
				9		MNP	
Melloni 2013^38^	LTM	heartbeat detection	proximal	10	40.55 yrs 55% F	Vipassana (4.35 yrs)	No
				10		MNP	
Nielsen 2006^39^	LTM	heartbeat detection	proximal	10	53.64 yrs 88% F	Vipassana/Zen (16.44) yrs	No
				16		MNP	
Otten 2015^29^	LTM	heartbeat tracking	proximal	22	39.60 yrs 55% F	MM (10.4 yrs)	No
				22		MNP	
Sze 2010^30^	LTM	heartbeat arousal	distal	21	29.35 yrs 64% F	Vipassana (7.1 yrs)	Yes
		skin conductance arousal	distal	21		MNP	
Xu 2018^40^	LTM	proprioceptive drift	distal	15 15	36.67 yrs 40% F	MM MNP	No

Note: Significant result found = statistically significant association between mindfulness and body awareness in original study; MM = Mindfulness Meditation; BS = Body Scan; FFMQ = Five-factor mindfulness questionnaire; SCS-M = Self-compassion scale, mindfulness subscale; MAAS = Mindful Attention Awareness Scale; proximal = direct comparison of bodily signal; distal = indirect comparison with bodily signal; LTM = long-term meditator; NA = not applicable. MNP = meditation naïve participant; Subject demographics yrs = age in years; % F = percentage female; Mindfulness type/control yrs = years of practice for LTMs. *Cebolla *et al*.^42^ included effect sizes for both LTM and trait designs.

### Study characteristics

Participants in the sample were on average 38.46 years old (*SD* = 13.88) and 66.3% female. The largest percentage of trials was conducted in the United States (41.2%) followed by Germany (23.5%) and the United Kingdom (17.7%).

Of the 17 samples, two^25,26^ examined the relationship between trait mindfulness and objective measures of body awareness, seven^26–28,31,34,35^ were randomized controlled trials (RCTs) assessing brief mindfulness training, and eight^29,30,35–40^ compared long-term practitioners with meditation naïve controls. Trait mindfulness was assessed using the Five Facet Mindfulness Questionnaire (FFMQ; *k* = 1) or Mindful Attention Awareness Scale (MAAS) and Self-Compassion Scale-Mindfulness^41^ (*k* = 1). One study^26^ contributed both an RCT and a trait mindfulness sample. While the originally reported analysis using trait mindfulness also included the RCT subsample, the authors were contacted and provided results from the trait mindfulness analysis with the overlapping portion removed.

Within the RCTs, mindfulness interventions ranged in length from a brief (e.g., ten minute) body scan induction^34^ to a 9-month mindfulness training^27^. To avoid duplicating data from the control condition in one study (and thereby violating assumptions of non-independence), only data from the two cohorts who completed the full 9-month mindfulness training and the retest control were included (i.e., a 3-month mindfulness condition was omitted). One study^35^ reported RCTs conducted on two independent samples. The average intervention length was 9.01 weeks (*SD* = 13.68), and four of seven interventions used active controls.

Long-term meditators (LTMs) reported practicing Vipassana or Zen an average of 9.38 years (*SD* = 4.04), although two studies did not report practice length for LTMs. One study^42^ included LTMs but also examined trait mindfulness in the same sample. For estimates of the overall omnibus effect, results from trait analyses were excluded to avoid non-independence among the included effect sizes.

**Figure 2:**
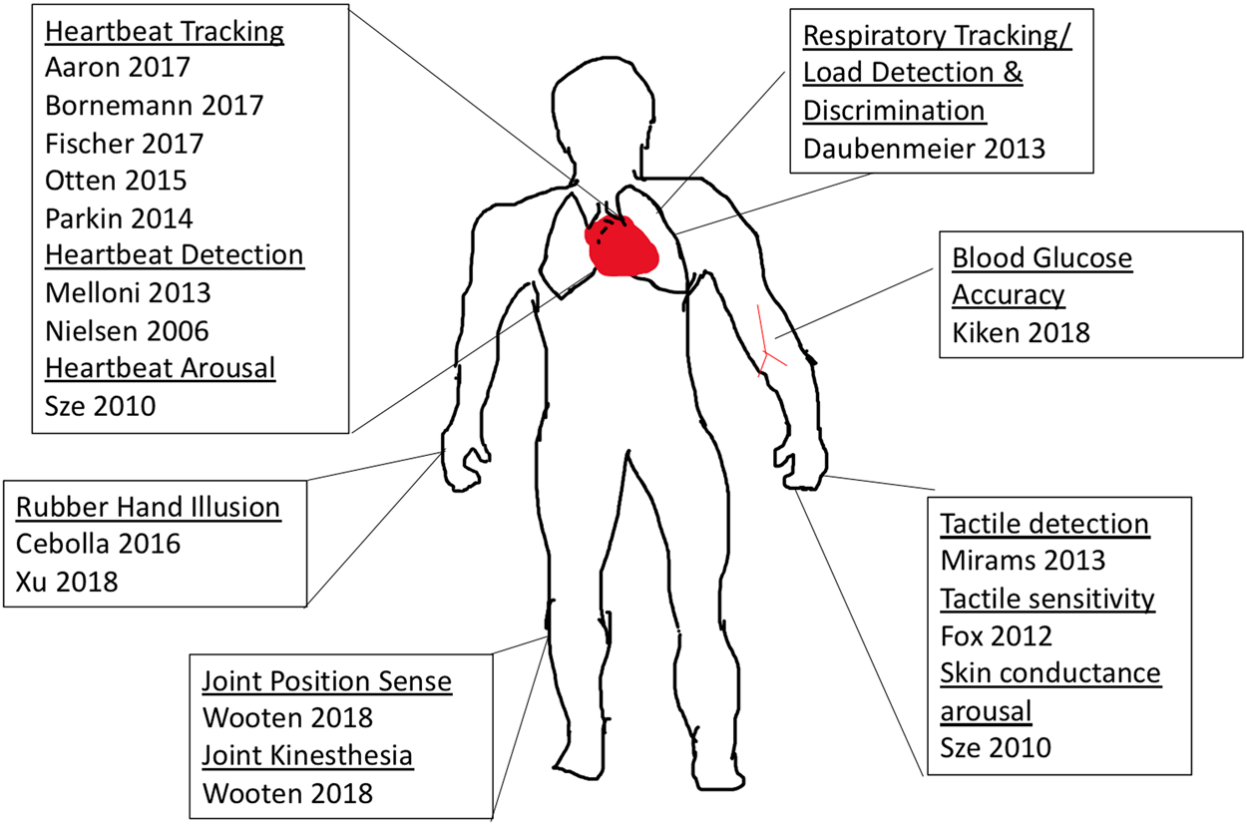
Measures and body location. Note that locations are used for illustration only and are approximate representations. See Table 2 for descriptions of tasks.

Objective measures of body awareness included measures of visceral (e.g., heartbeat), somatosensory (e.g., tactile sensitivity), and proprioceptive signals (e.g., joint position; see Fig. 2 and Table 2). The measures were categorized as proximal or distal measures of body awareness accuracy, based on whether they directly or indirectly compared participant report to the objective measure (see Methods). Eleven of the seventeen samples assessed body awareness proximally, two assessed body awareness proximally and distally, and the remaining four only contained distal measures of body awareness (Table 1 and Supplemental Materials Table 1). The majority of body awareness tasks (53.3%) consisted of heartbeat tasks (i.e., detection or tracking).

**Table 2:** Description of body awareness tasks.

Task Name	Task Description	Study
Blood Glucose Accuracy	Participants report blood glucose levels, compared with actual levels.	Kiken 2018^25^
Heartbeat Arousal	Participants report arousal levels every minute, compared with heartbeat to calculate cross-correlation.	Sze 2010^30^
Heartbeat Detection	Tones are played either concurrently or non-concurrently with heartbeat, participants detect when concurrent.	Nielsen 2006^39^, Melloni 2013*^38^
Heartbeat Tracking	Participants count their heartbeats over a short period, and then report total at the end. Total counted is compared to actual.	Parkin 2014^26^, Otten 2015^29^, Aaron 2017^34^, Bornemann 2017^27^, Fischer 2017^35^
Joint Kinesthesia	Participants knee joints are moved until the participant detects movement. Degrees of movement is reported.	Wooten 2018^28^
Joint Position Sense	Participants knee joints are moved through a range of angles. The participant reports when their knee joint reaches a target angle. The difference between the actual angle and target angle is computed.	Wooten 2018^28^
Proprioceptive Drift	Participant’s proprioceptive mapping is assessed with the rubber hand illusion (Botvinick & Cohen, 1998)^87^	Cebolla 2016^42^, Xu *et al*. 2018^40^
Respiratory Load Detection	Participants breath through a tube, and various levels of resistance are put in the air flow. Participants detect whether or not resistor is present.	Daubenmeier 2013^36^
Respiratory Load Discrimination	Participants breath through a tube, and various levels of resistance are put in the air flow. Participants rate the level of resistance, correspondence is quantified.	Daubenmeier 2013^36^
Respiratory Tracking	Participants move dial to indicate breath depth. Tidal volume of breath is measured. Cross-correlation of dial ratings and tidal volume is calculated.	Daubenmeier 2013^36^
Skin Conductance Arousal	Same as Heartbeat Arousal, but skin conductance and other physiological measures are compared with arousal rating.	Sze 2010^30^
Tactile Detection**	Participants receive vibrating stimulus on left index finger at the threshold of detection. Report yes or no for presence of stimulus.	Mirams 2013^31^
Tactile Sensitivity	Participants rate tactile sensitivity of their body parts. Ratings are compared to literature data collected by two-point discrimination.	Fox 2012^37^

*Melloni *et al*.^38^ conducted heartbeat detection variant, they had participants tap a mouse for every heartbeat registered.

**Mirams *et al*.^31^ report signal detection measures. In our quantitative synthesis we use sensitivity or *d’*, as it is a special case of accuracy (with bias removed).

### Risk of bias within studies

Supplemental Materials Table 2 presents results from the modified Jadad study^43^ quality coding. Of note, Jadad coding was conducted for all studies, although it is perhaps most relevant for interpreting the quality of the randomized designs. All studies included an objective measure of body awareness that was considered blind to group assignment. A minority (41.2%) of studies used a randomized design. Modified Jadad scores ranged from one to four, with a mean of 2.00 (*SD* = 1.12). Three studies^27,28,34^ received a score of one across on all four Jadad items.

### Results of individual studies

**Figure 3:**
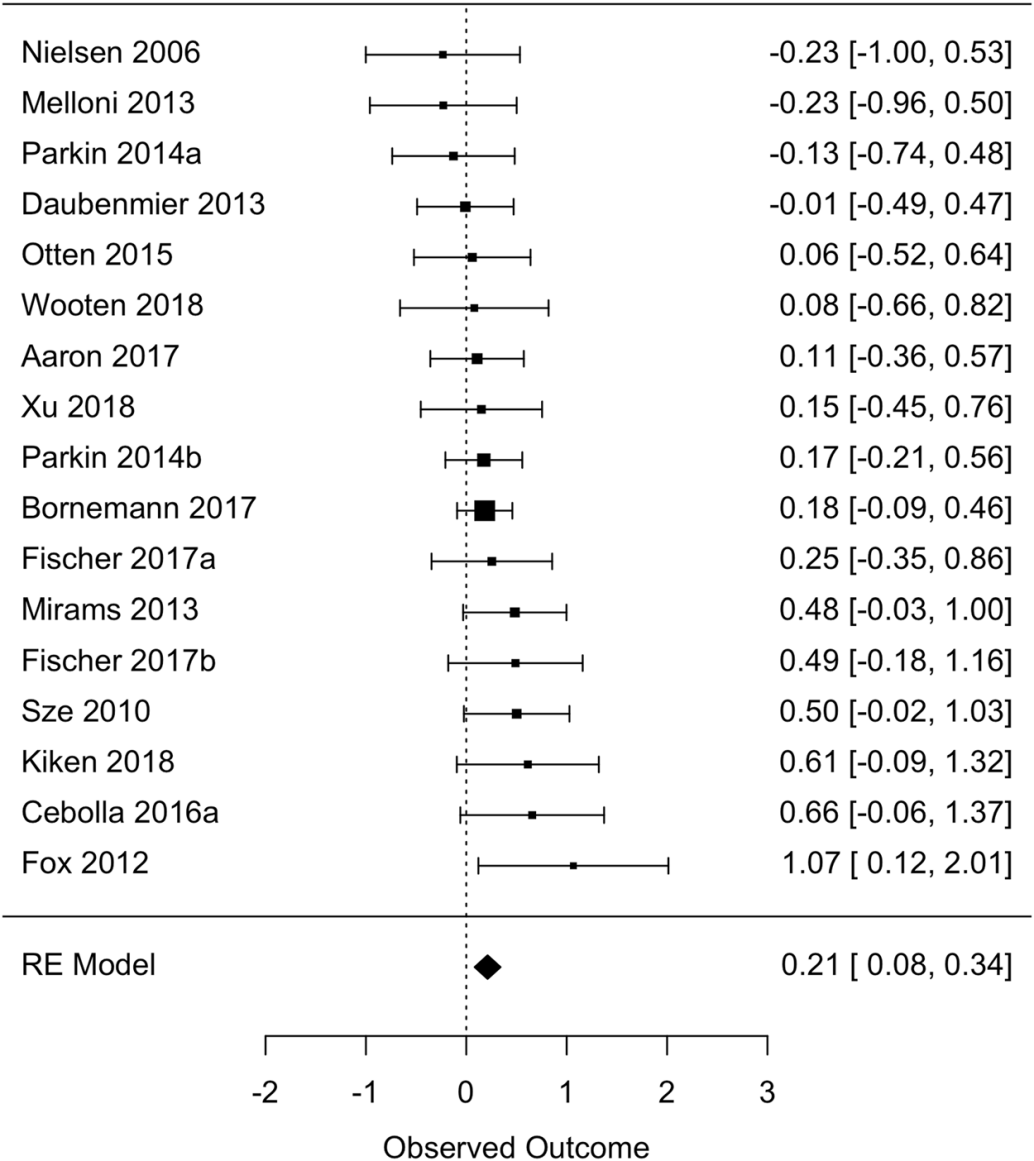
Forest plot of study-level and overall omnibus effect sizes in Cohen’s *d* units. Size of points reflect the weight of each study in the meta-analysis (i.e., the inverse of the variance of the effect sizes). Error bars represent 95% confidence interval^79^.

Study-level effect sizes are reported in Fig. 3.

### Synthesis of results

An overall omnibus effect size was computed by aggregating across all three study designs (Table 3). Across 17 independent effect sizes, a small but statistically significant effect was detected (*g* = 0.21, 95% CI [0.08, 0.34]) indicating a positive relationship between mindfulness and objective measures of body awareness. Heterogeneity was low, although with a wide confidence interval (*I*^2^ = 0.0%, [0.0, 63.0]).

**Table 3:** Meta-analytic results.

Model	*k*	*n*	*ES* [95% *CI*]	*I*^2^ [95% *CI*]	*k*_imp_	*ES*_adj_ [95% *CI*]	*FSN*
Overall	17	879	0.21 [0.08, 0.34]	0.0 [0.0, 63.0]	0	NA	53
Trait	3	160	0.24 [−0.06, 0.55]	0.0 [0.0, 97.0]	0	NA	1
LTMs	8	244	0.20 [−0.04, 0.44]	13.5 [0.0, 86.9]	0	NA	3
RCTs	7	505	0.20 [0.02, 0.38]	0.0 [0.0, 68.9]	0	NA	5
Proximal	13	759	0.17 [0.03, 0.31]	0.0 [0.0, 55.5]	0	NA	10
Distal	6	170	0.31 [0.01, 0.61]	18.5 [0.0, 91.4]	1	0.24 [−0.08, 0.56]	7
Heartbeat	9	622	0.13 [−0.03, 0.29]	0.0 [0.0, 58.1]	2	0.16 [0.01, 0.32]	0

Note: *k* = independent samples included; *n* = total sample size; *ES* = effect size in Hedges’ *g* units; *CI* = confidence interval; *I*^2^ = between-study heterogeneity; *k*_imp_ = number of studies imputed to account for asymmetric funnel plot; *ES*_adj_ = adjusted effect size based on trim-and-fill analysis; *FSN* = fail-safe N; NA = not applicable due to insufficient studies or absence of imputed studies; Proximal = omnibus effect size computed using proximal measures of body awareness only; Distal = omnibus effect size computed using distal measures of body awareness only.

A formal test of moderation did not detect differences between the three study designs in the magnitude of the relationship between mindfulness and body awareness (*Q*[2] = 0.15, *p* = 0.926). However, when examined separately by study design, mindfulness remained associated with body awareness only within the seven RCTs^26–28,31,34,35^ (*g* = 0.20, [0.02, 0.38]). Heterogeneity across RCTs was low, although again with a wide confidence interval (*I*^2^ = 0.0%, [0.0, 68.9]). Mindfulness was not associated with body awareness in three studies examining the relationship between trait mindfulness and body awareness^25,26,41^ (*g* = 0.24, [−0.06, 0.55], *I*^2^ = 0.0%, [0.0, 97.0]) or eight studies comparing LTMs with controls^29,30,35–40^ (*g* = 0.20, [−0.04, 0.44], *I*^2^ = 13.5%, [0.0, 86.9]).

Omnibus effects were also estimated restricting to either proximal or distal measures of body awareness. Across 13 studies^25–29,31,34–36,38,39^, a small but statistically significant relationship was detected among proximal measures (*g* = 0.17, [0.03, 0.31], *I*^2^ = 0.0%, [0.0, 55.5]). Across six studies^28,30,35–37,40,42^, a significant relationship was also detected among distal measures (*g* = 0.31, [0.01, 0.61], *I*^2^ = 18.5%, [0.0, 91.4]). Whether body awareness was assessed using a proximal or distal measure did not significantly moderate effect sizes (*Q*[1] = 0.75, *p* = 0.387).

An omnibus effect size was also computed restricting to heartbeat-related tasks (tracking or detection). Across nine studies^26,29,30,34,35,37–39^, mindfulness was not associated with heartbeat-related tasks (*g* = 0.13 [−0.03, 0.29], *I*^2^ = 0.0 [0.0, 58.1]).

### Risk of bias across studies

Bias across studies was assessed through funnel plots, trim-and-fill adjusted analyses, calculation of fail-safe N, and a moderator test examining the association between modified Jadad scores and effect sizes. Evidence for publication bias was found when examining distal measures and heartbeat-related tasks (Table 3). For distal measures, the previously significant effect was no longer significant (*g* = 0.24, [−0.08, 0.56]). In contrast, for heartbeat-related tasks, trim-and-fill adjustment imputed two studies on the right side (i.e., studies with positive effect sizes), yielding an adjusted effect size that did differ from zero (*g* = 0.16 [0.01, 0.32]). Study quality was not associated with effect sizes in the omnibus analysis (*Q*[1] = 0.01, *p* = 0.943). Fail-safe Ns were generally very small (0 to 53; Table 3), and all failed to meet the recommended cut-off (i.e., fail-safe N > 5*n + *10, where *n* = the number of published studies^44^.

## Discussion

The current study meta-analytically examined the relationship between mindfulness – operationalized as a dispositional trait and short- (RCTs) or long-term (LTM) training in mindfulness meditation– with objective measures of body awareness. Our findings indicate a small but statistically significant positive relationship between mindfulness and objective measures of body awareness (*g* = 0.21). The relationship was largely homogeneous across studies (although with a wide confidence interval for estimates of heterogeneity). Effect sizes were similar in magnitude across all three study designs and study design did not statistically moderate effects. However, when examined separately, mindfulness remained associated with body awareness only among RCTs, potentially due to the considerably larger sample size associated with this analysis (*n* = 505). Among studies using trait mindfulness or LTMs, no significant associations were found, perhaps due to the combination of small omnibus effect sizes and small sample sizes for these analyses. Effects appeared similar across both proximal and distal measures of body awareness. Effects were also not significant when examined among heartbeat-related measures alone (which is consistent with another recent meta-analysis, restricted to heartbeat measures in long-term meditators^45^), although this effect became significant following a trim-and-fill analysis adjusting for potential publication bias. Study quality did not predict variation in effect sizes, although overall study quality was modest (Jadad score = 2.00 out of 4.00).

While supporting the possibility of a link between mindfulness and body awareness, results should be interpreted cautiously. While a trim-and-fill analysis of the omnibus effect did not indicate publication bias through asymmetric funnel plots, potential publication bias was evident in a trim-and-fill analysis restricted to distal measures (as well as for heartbeat tasks, although in the opposite direction, i.e., unpublished positive findings). Estimates of the fail-safe N suggest that results are highly tenuous and not robust to threats of publication bias. Relatedly, the total number of samples (*k* = 17) and participants (*n* = 879) were modest.

What can be made of the small but statistically significant effects observed in the current meta-analysis? Findings, in particular from the seven included RCTs, provide, to our knowledge, novel meta-analytic evidence supporting a relationship between training in mindfulness and an objective behavioral measure. These results are notable, given mixed findings on the relationship between mindfulness training and other objective behavioral measures (e.g., executive functioning^46,47^). Importantly, the objective measures of body awareness included in the current review are by nature less susceptible than self-report measures (e.g., of psychological symptoms, well-being) to response set biases that have long been raised as criticism of the mindfulness literature^48–50^ and measurement broadly^51^.

The potential novelty of this finding is qualified by the modest effect sizes not robust to the possibility of publication bias based on failsafe-N estimates. It appears that mindfulness may be only weakly associated with body awareness accuracy (i.e., the correct and precise monitoring of bodily sensations). A primary reason for this may be the kind of attention that is typically trained through mindfulness. In contrast to other forms of attentional training that direct attention to a single stimulus^52^, mindfulness as traditionally taught (e.g., in MBSR) involves attending to a more diffuse array of stimuli. Even training specifically in body awareness (e.g., body scan) involves moving the attention systematically throughout the body, attending to a variety of bodily sensations (e.g., tingling, pressure, heat^53^). Mindfulness practice may change one’s relationship with bodily sensations (e.g., shifted appraisals^6,54^), with ramifications for emotions, decision-making, and general well-being^55^, even in the absence of improving body awareness accuracy.

The modest relationship detected here may represent body awareness not focused on a particular type of sensation. Consider, for example, heartbeat tasks, varieties of which were included in nine of the 17 studies in this meta-analysis. Mindfulness practices do not involve any particular emphasis on the sensations associated with one’s heartbeat, which could explain the weak and statistically non-significant association detected using this task (*g* = 0.13). Mindfulness may instead change more distal processes (e.g., using detection of heartbeats to appraise one’s general arousal), as was found in one of the included studies^30^. In this study, long-term mindfulness meditation practitioners rated their arousal to video stimuli, while investigators measured heartbeat. Meditators showed significantly larger cross-correlations (i.e., coherence^56^) between arousal ratings and actual heartbeat than controls. Other findings suggest that task-specific improvements in body awareness are more likely within task-focused training paradigms^57–59^.

While results suggest that mindfulness only modestly relates to body awareness (if at all), clinical trials suggests that mindfulness-based interventions are associated with symptom improvement for conditions marked by body awareness dysfunction. One potential explanation for this is that mindfulness is associated with some aspects of body awareness and not others. Body awareness has been theorized to include two relatively independent axes: accuracy and sensibility. As described previously, accuracy involves the correct and precise monitoring of bodily sensations and was the focus of the current meta-analysis. Sensibility, in contrast, is a broader construct which captures a participant’s subjective experience of bodily sensations^60^. Sensibility can be measured via self-report assessments like the Multidimensional Assessment of Interoceptive Awareness [MAIA], containing dimensions like non-distracting (i.e., a reduced tendency to ignore or distract oneself from uncomfortable bodily sensations)^33^.

Each of these two axes of body awareness – sensibility and accuracy – appear to have value in understanding psychopathology^54,61^. Body awareness accuracy below a certain threshold may explain specific types or degrees of dysfunction. For example, alexithymia is characterized by very low body awareness accuracy^62,63^, which could explain why participants suffer deficits in emotional processing^64^. However, when body awareness accuracy is at or above a threshold, body sensibility may become more relevant to psychopathology and well-being. An example is somatic anxiety, where patients have high accuracy^65^, but harmful body sensibility (e.g., repeated habits of pain catastrophizing^61^.

Mindfulness-based interventions may support well-being by subtly adjusting both of these axes of body awareness. Mindfulness may improve body awareness accuracy slightly, with potentially large benefits for patients with more highly impaired accuracy (e.g., those with alexithymia). But for others, the benefits of mindfulness may come from changes in body awareness sensibility (e.g., reducing habits of pain catastrophizing). More empirical work is needed to further map the relationship of mindfulness with these two axes of body awareness.

The current meta-analysis implies several directions for future research. One key future direction is examining the degree to which intervention-related improvements in symptoms linked to interoceptive awareness (e.g., chronic pain) are mediated by changes in objective measures of body awareness. Based on the theoretical possibility that mindfulness may improve domain-general (rather than domain-specific) body awareness, it may be valuable to assess body awareness through multiple measures. In addition, structural and functional neuroimaging studies could examine the neural bases of body awareness (e.g., intervention-dependent plasticity in the insula^6,24^) as predictors of intervention effects. Lastly, body awareness could be examined at baseline and used as a predictor (i.e., moderator) of treatment response, based on the possibility that individuals with low body awareness may benefit most from training in this capacity.

### Study limitations

Our conclusions were limited by the relatively small sample of studies and small overall number of included participants. Research examining mindfulness and body awareness is nascent, with the earliest study included in our meta-analysis from 2006. Another limitation was heterogeneity across study designs. While we report both an overall omnibus effect size as well as effect sizes across different subgroupings of studies (i.e., separated by study design, proximal vs. distal measures, heartbeat tasks), there remained variation across studies that was not modeled. For example, the length of training varied across the seven included RCTs, which makes interpretation of the overall effect more ambiguous (although a *post hoc* analysis indicated that length of training did not moderate effect sizes within RCTs, *Q*[1] = 0.01, *p* = 0.914). Similarly, degree of experience within and between the LTM samples varied (although a *post hoc* analysis indicated that years of experience for LTM samples also did not moderate effect sizes, *Q*[1] = 0.35, *p* = 0.555). LTMs likewise may have engaged in a variety of meditation practices that were not assessed. Other comparisons (e.g., relationship between trait mindfulness and body awareness) were almost certainly underpowered to detect a small effect (*k* = 3 studies, *n* = 160). Body awareness tasks themselves also varied, and only heartbeat-related tasks were examined separately due to the small number of studies using equivalent tasks (e.g., *k* = 1 study examining glucose monitoring^25^). Lastly, we restricted inclusion criteria to studies published in English. It is therefore possible that eligible studies were excluded, although only five studies were excluded for being published in a language other than English.

It is also possible that issues of reliability and validity related to objective measures of body awareness limited our results. While objective measurement was a strength, the included body awareness measures are not without criticism (e.g., heartbeat tracking can be confounded with time estimation ability and body mass index^66^).

These limitations notwithstanding, the current study is the first to meta-analytically examine the relationship between mindfulness and body awareness. The small but statistically significant effects detected in the overall sample and among RCTs specifically supports further work seeking to delineate this relationship.

## Methods

### Objectives

The current meta-analysis sought to clarify the relationship between mindfulness and objective measures of body awareness. The Preferred Reporting Items for Systematic Reviews and Meta-Analyses (PRISMA) Standards were followed^67^.

### Protocol and registration

No previously published protocol nor pre-registration exists for this meta-analysis.

### Eligibility criteria

Studies were eligible if they included an objective measure of body awareness examined in relationship to mindfulness. We operationalized mindfulness to encompass three distinct study designs: (a) associations between self-reported trait mindfulness and measures of body awareness, (b) randomized controlled trials (RCTs) comparing mindfulness training to a control condition, and (c) comparisons between long-term mindfulness meditation practitioners (LTMs) and meditation naïve controls. For studies examining trait mindfulness, mindfulness must have been assessed with validated measures purported to measure mindfulness as construed within MBSR and similar interventions (e.g., mindfulness-based cognitive therapy [MBCT]^68^. One example is the MAAS, which assesses an individual’s tendency for mindful states, in particular the frequency of present-centered attention/awareness^3^. RCTs had to include random assignment of participants to training in mindfulness meditation practices (e.g. MBSR). MBSR is a prototypical mindfulness-based intervention that is eight-weeks in length and involves instruction in mindfulness practices (e.g., body scan and breath awareness^69^). MBSR is traditionally delivered in a group setting and also includes home meditation practice^69^. For studies involving LTMs, meditation experience must have primarily included Buddhist contemplative practices from which secular mindfulness was derived (i.e., Theravadin/Vipassana, Zen^70^. Eligible studies referred to their sample of long-term meditation practitioners as “expert” or “long-term meditators.”

The literature on body awareness and the related construct, interoception, was reviewed to determine a definition for objective measures of body awareness. Broadly, body awareness encompasses noticing, identifying, experiencing, and attending to bodily sensations^71^. Bodily sensations may be caused by internal or external conditions^72^, and include temperature, pain, itch, tickle, muscular and visceral sensations, vasomotor flush, hunger, and thirst^73^ For the purposes of this meta-analysis, we adopted a definition of body awareness encompassing visceral, somatosensory and proprioceptive (i.e., position of the body in space^74^) signals. Thus, our definition overlaps with interoception, commonly considered to be the processing of internal bodily signals^32^.

### Outcomes of interest

To determine whether a particular measure could be viewed as an objective measure of body awareness, we use a framework applied to interoception by Khalsa and Lapidus^32^. Khalsa and Lapidus define interoceptive accuracy as “correct and precise monitoring” (p. 2) of bodily sensations. Interoceptive accuracy measures include tasks like heartbeat tracking, where a correspondence between counted heartbeats and objective count is assessed^75^. Recent investigations have determined accuracy measures and self-report measures capture almost entirely independent dimensions of interoception^60^.

Thus, for the purposes of the current study, we employed Khalsa and Lapidus’^32^ definition (correct and precise monitoring) to operationalize body awareness tasks. Specifically, eligible studies must assess how correctly and precisely participants monitor bodily sensations –including visceral (e.g., heartbeat), somatosensory (e.g., touch), or proprioceptive (e.g., position in space) sensations.

No restrictions were placed on the body system being assessed (e.g., heartbeat, respiration), publication status (i.e., published and unpublished studies were both eligible), publication date (i.e., databases were searched since inception), sample size, or target population (e.g. clinical or non-clinical). Due to resource limitations to support translation of potential studies, studies were restricted to those published in English. Only studies with adult participants (average age 18 years or older) were included.

Studies were excluded if they did not include a measure that assessed body awareness accuracy and mindfulness operationalized in one of the three ways described above (i.e., trait mindfulness, RCT, LTM). Studies were also excluded if they did not report data necessary for computing standardized effect sizes.

### Information sources

Studies were identified by searching four databases: PubMed, ISI Web of Science, PsycINFO, and Scopus. Databases were identified based on recent comprehensive reviews of mindfulness-based interventions^14,76^.

### Search

An initial search was performed on October 6^th^, 2018. The full search string was: (interocept* OR “bodily awareness” OR “body awareness” OR “somatic awareness” OR somatosensory OR visceral OR proprioception) AND (mindfulness OR meditation). The initial search was updated on November 4^th^, 2018. In the second search, we added terms related to specific measures of body awareness uncovered during the initial search. Additional terms were: (heat OR cold OR tactile OR “two-point discrimination”) AND (mindfulness OR meditation). The exact search strategy for each database is reported in Supplemental Materials.

### Study selection

Eligibility was independently assessed by both the first and second author, with disagreements discussed with the senior author. Studies were initially evaluated at the level of title and abstract and later as full manuscripts (Fig. 1). In two instances, the authors of the original studies were contacted for clarification relating to inclusion and exclusion criteria for the meta-analysis.

### Data collection process

A coding manual was developed by the first and senior author to guide the extraction of study descriptive and effect size data. Extraction of these data were conducted by the first and senior author and confirmed by the second author. Coding disagreements were discussed by the team.

### Data items

Study descriptive characteristics and data necessary for computing standardized effect sizes were extracted (Table 1). These included study design (i.e., trait mindfulness, RCT, LTM), operationalization of mindfulness, measure of body awareness, sample size, participant demographics, and description of intervention and comparison conditions. Quantitative data for computing effect sizes varied depending on the study design. For trait mindfulness, correlation coefficients between self-reported mindfulness and measures of body awareness were extracted, when available. For RCTs, pre- and post-test means and standard deviations were extracted. For LTMs, means and standard deviations for the LTM and control group were extracted.

Body awareness tasks were also coded as proximal or distal measures of body awareness. Tasks which directly compared participant’s self-report and a bodily signal were considered proximal, in keeping with Garfinkel *et al*.’s^50^ definition of interoceptive accuracy. For example, heartbeat tracking is a proximal task as it directly compares participants’ counted heartbeats to objective heartbeat. However, other tasks were deemed relevant, but involved an indirect measure of interoceptive accuracy. For example, Fox *et al*.^37^ compared subjective sensitivity across body regions to objective standards of tactile sensitivity (the 2-point discrimination threshold for each body region based on prior studies). Therefore, they assessed whether participants’ subjective hierarchies of sensation vividness matched objective hierarchies of sensory accuracy. Based on the perspective that a hierarchy of 2-point discrimination thresholds reflects an objective indicator, the concordance between participants’ subjective experience of sensitivity and these thresholds was deemed a measure of ‘correct and precise monitoring,’ consistent with our inclusion criteria. As Fox *et al*.^37^ used prior studies to establish a standardized hierarchy of tactile sensitivity instead of directly comparing a participant’s rating with a bodily signal, this task was coded as a distal measure.

### Risk of bias of individual studies

Risk of bias was assessed using a modified set of the Jadad criteria^43^ previously employed to assess mindfulness-based interventions^14,69^. Specifically, we coded (a) whether a trial was randomized, (b) whether the procedure for randomization was described and appropriate, (c) whether outcome assessment was blind to group assignment, and (d) whether reasons were given for dropouts and withdrawals. Items were coded as “yes” if present, “no” if not present, and “unclear” if presence was ambiguous. To compute the Jadad score, a sum of “yes” responses was computed across the four items. In order to assess the relationship between study quality and outcome, Jadad scores were examined as moderators of treatment effects in meta-regression models.

Consistent with Piet and Hougaard^77^ five additional items were also coded including (a) whether treatment allocation was concealed, (b) whether groups were similar at baseline, (c) whether the number of withdrawals and dropouts in each group were mentioned, (d) whether analyses were conducted using the intent-to-treat sample, and (e) whether a power analysis was reported.

### Summary measures

Calculation of standardized effect sizes reflecting the relationship between mindfulness and objective measures of body awareness varied by study design.

For trait mindfulness studies, correlation coefficients were used, when available. For trait studies that did not report this value, the closest approximation was used (e.g., difference in trait mindfulness for individuals with accurate vs. inaccurate assessment of blood glucose level^25^). To allow comparison and pooling with between-group effect sizes, correlations were converted into Cohen’s^78^*d* units using standard methods^79^.

For RCTs, consistent with other recent meta-analyses^14,80^ a pre-post effect size (i.e., Cohen’s *d*) was first computed for the mindfulness and control conditions separately using means and standard deviations. Unlike effect sizes based on only post-treatment data, this method accounts for potential baseline differences between groups^81^. A conservative estimate of the correlation between time points (*r* = 0.50) was assumed, given this correlation is not typically reported^82^. A between-group effect size on the metric of Cohen’s *d* was then computed by taking the difference between these within-group pre-post effect sizes, with the variance of the between-group *d* computed as the sum of the variance of the within-group effects^83^.

For LTM studies, an independent groups Cohen’s *d* effect size was computed using means and standard deviations based on standard formulas^81^.

Lastly, in order to account for potential upward small sample bias, all effect sizes were converted from Cohen’s *d* into Hedges’ *g* using standard formulas^71^. All analyses were conducted in the R statistical software environment using the ‘metafor,’ ‘MAc, and ‘MAd’ packages^83^.

### Synthesis of results

When studies included multiple effect sizes (i.e., multiple objective measures of body awareness), these were first aggregated within each study using the ‘MAd’ package. In order to most comprehensively characterize the included studies, all reported effects that met the inclusion criteria were included. Aggregating within study first assured that studies with multiple measures (or multiple methods of scoring a single measure) did not carry undue weight in the omnibus effect size estimates. For each study, an overall effect size in Hedges’ *g* units along with a 95% confidence interval (CI) was computed.

Omnibus estimates were computed in several ways to address various questions of interest. A single overall estimate aggregated effect sizes across all three study designs to address the broad question of whether mindfulness is associated with objective measures of body awareness. A formal test of moderation examined whether the strength of this relationship varied by study design (i.e., trait, RCT, LTM). Separate omnibus analyses also examined the relationship between mindfulness and body awareness for the three study designs, to assess whether mindfulness operationalized in these three ways related independently to body awareness. For all omnibus estimates, an effect size and 95% CI along with an estimate of between-study heterogeneity (*I*^2^) was computed. Heterogeneity was interpreted based on Higgins, Thompson, Deeks, and Altman’s^84^ guidelines. Analyses used random effect models with study effect sizes weighted by the inverse of their variance^85^.

One study, Cebolla *et al*.^42^ reported the relationship between trait mindfulness and LTM status with objective body awareness within the same sample of participants. To avoid including this sample twice, only data pertaining to the LTM comparison was included in the overall omnibus effect size. For analyses separated by design (i.e., trait mindfulness, LTM), both effect sizes from Cebolla *et al*. were included.

In addition, a set of models were run restricted to either proximal or distal measures of body awareness. A formal test of moderation examined whether the relationship between mindfulness and body awareness differed between proximal and distal measures.

Lastly, an omnibus effect size was also computed using only heartbeat-related tasks (e.g., heartbeat tracking, heartbeat detection), given these tasks have appeared repeatedly in the literature.

### Risk of bias across studies

Publication bias was assessed by visually inspecting funnel plots for asymmetry both in the overall sample as well as in sub-analyses (e.g., separately by study design). Models were also re-estimated using trim-and-fill methods to account for asymmetric distribution of effect sizes around the omnibus effect^85^. Meta-regression was used to examine the relationship between study quality (i.e., modified Jadad score) and treatment effects. A fail-safe N was also computed based on Rosenthal’s^86^ method reflecting the number of unpublished null findings that would need to exist to nullify the observed effect.

## Supplementary information

Supplementary Information
