# Supplementary material for: The relationship between mindfulness and objective measures of body awareness: A meta-analysis
Source: Sci Rep. 2019 Nov 22;9:17386. doi: 10.1038/s41598-019-53978-6 (PMC6874545; doi:10.1038/s41598-019-53978-6)
Supplement: Supplementary file 1 — Supplementary Information [file 41598_2019_53978_MOESM1_ESM.pdf]

The relationship between mindfulness and objective measures of body awareness: A meta-analysis

Isaac N. Treves[1], Lawrence Y. Tello[3], Richard J. Davidson[2,4], & Simon B. Goldberg[2,5,*]

[1] Department of Brain and Cognitive Sciences, Massachusetts Institute of Technology, Cambridge, Massachusetts, USA
[2]Center for Healthy Minds, University of Wisconsin – Madison, Madison, WI, 53703, USA
[3]National Eye Institute, National Institutes of Health, Bethesda, MD, 20892, USA
[4]Department of Psychology, University of Wisconsin – Madison, Madison, WI, 53706, USA
[5]Department of Counseling Psychology, University of Wisconsin – Madison, Madison, WI, 53706, USA

Correspondence and request for materials should be addressed to S.B.G. (email: sbgoldberg@wisc.edu)

PRISMA 2009 Checklist

Section/topic	#	Checklist item	Reported on page #
**TITLE**			
Title	1	Identify the report as a systematic review, meta-analysis, or both.	1
**ABSTRACT**			
Structured summary	2	Provide a structured summary including, as applicable: background; objectives; data sources; study eligibility criteria, participants, and interventions; study appraisal and synthesis methods; results; limitations; conclusions and implications of key findings; systematic review registration number.	1
**INTRODUCTION**			
Rationale	3	Describe the rationale for the review in the context of what is already known.	1-2
Objectives	4	Provide an explicit statement of questions being addressed with reference to participants, interventions, comparisons, outcomes, and study design (PICOS).	2
**METHODS**			
Protocol and registration	5	Indicate if a review protocol exists, if and where it can be accessed (e.g., Web address), and, if available, provide registration information including registration number.	7
Eligibility criteria	6	Specify study characteristics (e.g., PICOS, length of follow-up) and report characteristics (e.g., years considered, language, publication status) used as criteria for eligibility, giving rationale.	7
Information sources	7	Describe all information sources (e.g., databases with dates of coverage, contact with study authors to identify additional studies) in the search and date last searched.	8
Search	8	Present full electronic search strategy for at least one database, including any limits used, such that it could be repeated.	8, Supp Materials
Study selection	9	State the process for selecting studies (i.e., screening, eligibility, included in systematic review, and, if applicable, included in the meta-analysis).	8
Data collection process	10	Describe method of data extraction from reports (e.g., piloted forms, independently, in duplicate) and any processes for obtaining and confirming data from investigators.	8
Data items	11	List and define all variables for which data were sought (e.g., PICOS, funding sources) and any assumptions and simplifications made.	8-9
Risk of bias in individual studies	12	Describe methods used for assessing risk of bias of individual studies (including specification of whether this was done at the study or outcome level), and how this information is to be used in any data synthesis.	9
Summary measures	13	State the principal summary measures (e.g., risk ratio, difference in means).	9-10
Synthesis of results	14	Describe the methods of handling data and combining results of studies, if done, including measures of consistency (e.g., I^2) for each meta-analysis.	10

PRISMA 2009 Checklist

Section/topic	#	Checklist item	Reported on page #
Risk of bias across studies	15	Specify any assessment of risk of bias that may affect the cumulative evidence (e.g., publication bias, selective reporting within studies).	10
Additional analyses	16	Describe methods of additional analyses (e.g., sensitivity or subgroup analyses, meta-regression), if done, indicating which were pre-specified.	6
**RESULTS**			
Study selection	17	Give numbers of studies screened, assessed for eligibility, and included in the review, with reasons for exclusions at each stage, ideally with a flow diagram.	6, 22
Study characteristics	18	For each study, present characteristics for which data were extracted (e.g., study size, PICOS, follow-up period) and provide the citations.	2-3, 17-18
Risk of bias within studies	19	Present data on risk of bias of each study and, if available, any outcome level assessment (see item 12).	3, Supp Materials
Results of individual studies	20	For all outcomes considered (benefits or harms), present, for each study: (a) simple summary data for each intervention group (b) effect estimates and confidence intervals, ideally with a forest plot.	24
Synthesis of results	21	Present results of each meta-analysis done, including confidence intervals and measures of consistency.	3-4
Risk of bias across studies	22	Present results of any assessment of risk of bias across studies (see Item 15).	4
Additional analysis	23	Give results of additional analyses, if done (e.g., sensitivity or subgroup analyses, meta-regression [see Item 16]).	6
**DISCUSSION**			
Summary of evidence	24	Summarize the main findings including the strength of evidence for each main outcome; consider their relevance to key groups (e.g., healthcare providers, users, and policy makers).	4
Limitations	25	Discuss limitations at study and outcome level (e.g., risk of bias), and at review-level (e.g., incomplete retrieval of identified research, reporting bias).	4-6
Conclusions	26	Provide a general interpretation of the results in the context of other evidence, and implications for future research.	4-7
**FUNDING**			
Funding	27	Describe sources of funding for the systematic review and other support (e.g., supply of data); role of funders for the systematic review.	16

From: Moher D, Liberati A, Tetzlaff J, Altman DG, The PRISMA Group (2009). Preferred Reporting Items for Systematic Reviews and Meta-Analyses: The PRISMA Statement. PLoS Med 6(7): e1000097. doi:10.1371/journal.pmed1000097

For more information, visit: **www.prisma-statement.org**.

Supplemental Materials Table 1. List of distal tasks

**Task**	**Paper**
Proprioceptive drift	Cebolla 2016, Xu 2018
Tactile sensitivity	Fox 2012
Respiratory load detection, respiratory load discrimination	Daubenmeier 2013*
Heartbeat arousal, skin conductance arousal	Sze 2010
Joint kinesthesia	Wooten 2018*

Note: Tasks were defined as distal if they involved indirect measure of interoceptive accuracy (Garfinkel et al., 2015). All tasks fit inclusion criteria of 'correct and precise monitoring' (Khalsa & Lapidus, 2016). *These papers contained multiple body awareness accuracy measures, and reported effect sizes for proximal measures as well.

Supplemental Materials Table 2. Modified Jadad coding of study quality

AuthorYear	**Random-ized**	**Randomization described and appropriate**	Treatment allocation concealed	Groups similar at baseline	**Blind outcome assessment**	Number of with-drawals/dropouts in each group mentioned	**Reasons given for with-drawals/ dropouts**	Intent-to-treat analysis	Power calculation described	Jadad score
Aaron 2017	**Yes**	**Yes**	Unclear	Yes	**Yes**	No	**Yes**	No	No	4
Bornemann 2017	**Yes**	**Yes**	Unclear	Yes	**Yes**	Yes	**Yes**	No	No	4
Cebolla 2016a	**No**	**No**	No	No	**Yes**	Yes	**Yes**	Yes	No	2
Cebolla 2016b	**No**	**No**	No	No	**Yes**	Yes	**Yes**	Yes	No	2
Daubenmier 2013	**No**	**No**	No	No	**Yes**	Yes	**No**	Unclear	No	1
Fischer 2017a	**Yes**	**No**	Unclear	Yes	**Yes**	No	**No**	Unclear	No	2
Fischer 2017b	**Yes**	**No**	Unclear	Yes	**Yes**	No	**No**	Unclear	No	2
Fox 2012	**No**	**No**	No	No	**Yes**	No	**Yes**	No	No	2
Kiken 2018	**No**	**No**	No	No	**Yes**	No	**No**	Unclear	No	1
Melloni 2013	**No**	**No**	No	No	**Yes**	No	**No**	Unclear	No	1
Mirams 2013	**Yes**	**Yes**	Yes	Yes	**Yes**	No	**No**	Unclear	No	3
Nielsen 2006	**No**	**No**	No	No	**Yes**	Yes	**Yes**	No	Yes	2
Otten 2015	**No**	**No**	No	No	**Yes**	No	**No**	Unclear	No	1
Parkin 2014a	**Yes**	**No**	Unclear	Yes	**Yes**	No	**No**	Unclear	No	2
Parkin 2014b	**No**	**No**	No	No	**Yes**	No	**No**	Unclear	No	1
Sze 2010	**No**	**No**	No	No	**Yes**	No	**No**	Unclear	No	1
Wooten 2018	**Yes**	**Yes**	Unclear	Unclear	**Yes**	Yes	**Yes**	No	No	4
Xu 2018	**No**	**No**	No	No	**Yes**	No	**No**	Unclear	No	1

Note: Bolded columns used to compute modified Jadad score per Piet and Hougaard (2011). Responses coded as “yes” received 1 point and those coded as “no” received 0 points.

Supplemental Materials Table 3. Search strategy

Database	Search string	Search fields
PubMed	(interocept* OR "bodily awareness" OR "body awareness" OR "somatic awareness" OR somatosensory OR visceral OR proprioception OR heat OR cold OR tactile OR "two-point discrimination") AND (mindfulness OR meditation)	All fields
Scopus	(interocept* OR "bodily awareness" OR "body awareness" OR "somatic awareness" OR somatosensory OR visceral OR proprioception OR heat OR cold OR tactile OR "two-point discrimination") AND (mindfulness OR meditation)	Article title, abstract, keywords
Web of Science	(interocept* OR "bodily awareness" OR "body awareness" OR "somatic awareness" OR somatosensory OR visceral OR proprioception OR heat OR cold OR tactile OR "two-point discrimination") AND (mindfulness OR meditation)	All fields
PsycINFO	(interocept* OR "bodily awareness" OR "body awareness" OR "somatic awareness" OR somatosensory OR visceral OR proprioception OR heat OR cold OR tactile OR "two-point discrimination") AND (mindfulness OR meditation)	All fields

Note: All databases were search from inception. No additional exclusions were made within the search (i.e., dissertations were discoverable within PsycINFO).
